# Effect of Supracervical Apposition and Spontaneous Labour on Apoptosis and Matrix Metalloproteinases in Human Fetal Membranes

**DOI:** 10.1155/2013/316146

**Published:** 2013-09-11

**Authors:** Mahalia Chai, Susan P. Walker, Clyde Riley, Gregory E. Rice, Michael Permezel, Martha Lappas

**Affiliations:** ^1^Department of Obstetrics and Gynaecology, University of Melbourne and Mercy Perinatal, Mercy Hospital for Women, Level 4/163 Studley Road, Heidelberg, VIC 3084, Australia; ^2^Research Centre, Mercy Hospital for Women, Heidelberg, VIC 3084, Australia; ^3^UQ Centre for Clinical Research, The University of Queensland, Herston, QLD 4029, Australia

## Abstract

*Background*. Apoptosis and matrix metalloproteinase (MMP-9) are capable of hydrolysing components of the extracellular matrix and weakening the fetal membranes which leads to eventual rupture, a key process of human parturition. The aim of this study was to determine the effect of supracervical apposition and spontaneous labour on apoptosis and MMP-9 in human fetal membranes at term. *Methods*. Fetal membranes were obtained from term non-labouring supracervical site (SCS) and compared to (i) a paired distal site (DS) or (ii) site of rupture (SOR) after spontaneous labour onset. *Results*. The expression of the proapoptotic markers Bax, Smac, Fas, FasL, caspase-3, and PARP, was significantly higher in the non-labouring SCS chorion compared to paired DS. Bax, Smac, FasL, caspase-3, and PARP staining was higher in the non-labouring SCS fetal membranes than that in the post-labour SOR. MMP-9 expression and activity were higher in the post-labour SOR fetal membranes compared to non-labouring SCS fetal membranes. *Conclusion*. Components of the apoptotic signalling pathways and MMP-9 may play a role in rupture and labour. Non-labouring SCS fetal membranes display altered morphology and altered apoptotic biochemical characteristics in preparation for labour, while the laboured SOR displays unique MMP characteristics.

## 1. Introduction

Spontaneous rupture of the fetal membranes usually occurs during labour at term, but in 10% of births at term and 30% of preterm births, fetal membranes rupture prior to the onset of labour. These events are, respectively, termed prelabour rupture of the membranes (PROM) and preterm prelabour rupture of the membranes (PPROM). PPROM is the leading identifiable cause of preterm birth; one-third of the estimated 13 million preterm births each year are attributed to PPROM [1, 2]. The exact causes of PPROM remain poorly understood, but it is possible that the biochemical pathways leading to fetal membrane rupture occur, albeit too early. Therefore, to reduce the burden of prematurity related to PPROM, it is first necessary to understand the biological mechanisms responsible for fetal membrane rupture at term.

The physical forces of uterine contractions are neither necessary nor sufficient to cause membrane rupture alone. It has been theorised that fetal membranes must rupture in part due to a programmed remodelling and maturation process which weakens the fetal membranes [3]. Animal studies chronicle the gestational changes in fetal membranes [4–6]. A focal area of the human fetal membranes overlying the cervical os, the supracervical site (SCS), exhibits morphological changes which has been shown to encompass the site of rupture (SOR). In seminal studies, Bourne (1962) transcervically marked SCS during the early stages of labour [7]. In the patients that continued to deliver vaginally, the marked SCS was situated on the tear line of the spontaneously, post-labour SOR [7]. The SCS and the SOR are up to 50% physically weaker compared to the DS [8, 9]. The post-labour SOR has reduced decidua, and the fibroblast and spongy layers are more disorganised and have fewer collagenous fibres compared to more distal sites (DS) [10]. Similar morphological observations have been found at the SCS prior to labour [11], which are consistent with collagen remodelling and apoptosis [9, 11–15].

Apoptosis or programmed cell death is induced via two distinct but converging pathways: the extrinsic and intrinsic pathways; both pathways converge with the activation of the caspase family [14, 16–18]. The balance of pro- and antiapoptotic proteins determines the cells commitment to apoptosis [19]. Previous studies have found increased apoptotic bodies [20] and intrinsic pathway apoptosis [11] in the no-labour SCS compared to DS. Matrix metalloproteinases (MMPs) are a family of zinc-dependent enzyme endopeptidases, known to degrade components of the extracellular matrix (ECM) in the fetal membranes [15, 21, 22]. A complex balance between degradation and remodeling activity of the matrix components maintains the integrity of the membranes throughout gestation. An imbalance favouring the MMPs over inhibitors is associated with labour and fetal membrane rupture [15, 23]. A role for MMP-2 and MMP-9 in spontaneous rupture of membranes in term labour and PPROM and infection has been demonstrated [24–27]. Pro-MMP-9 has been found to temporally increase with labour in DS of term fetal membranes compared to no-labour DS fetal membranes [6, 23, 28]. The non-labouring SCS has increased MMP-9 production in fetal membranes, but no regional differences were found after labour [14]. 

Fetal membrane rupture, specifically the changes in the fetal membranes which result in localised weakening and degradation in what were previously intact tissues, is not comprehensively understood. It has been shown that apoptosis and collagen remodeling occur in the fetal membranes [4, 11, 29]; however, the question remains as to the biological occurrence of apoptosis and MMPs in the fetal membranes at the critical region of interest: the “no-labour” SCS and “post-labour” SOR. Hence, the aims of this study were to (1) compare the regionalised biochemical changes (apoptosis expression and MMP expression) that occur in the no-labour SCS compared to DS fetal membranes and (2) to characterise the effects of labour by comparing the non-labouring SCS compared to post-labour SOR fetal membranes. 

## 2. Methods

### 2.1. Sample Collection and Preparation

The Research Ethics Committee of Mercy Health approved this study. Written, informed consent was obtained from all participating women. Human placentae and attached fetal membranes were obtained from 2 groups of women who delivered singleton infants at term (≥37 weeks of gestation): (i) Group 1: “no-labour” where women were not in labour at elective caesarean section (indications for caesarean section were breech presentation and/or previous caesarean section) and (ii) Group 2: “after labour” where women had spontaneous labour and membrane rupture. Six patients were collected from each group. For Group 1, samples were obtained from the SCS and DS. Identification of the SCS was performed as we have previously detailed [11]. Briefly, Bonney's blue dye was introduced through the cervix prior to caesarean section. Upon delivery of the placenta, a blue mark was obvious on the chorion facing membrane where the dye had been applied. DS amnion and choriodecidua were obtained approximately 2 cm from the periplacental edge. For Group 2, amnion and chorion were obtained from the tear line as we have previously described [30]. Patients with complicated clinical histories, including diabetes and hypertension, were excluded. The clinical summary of patients used in this study is shown in Table 1. 

Herein, fetal membranes overlying the cervix from non-labouring placentas will be referred to as SCS and the membranes from the post-labour tear line will be referred to as SOR. Tissue samples were fixed in buffered formaldehyde solution (4%) and paraffin embedded for morphological and immunohistochemical analysis, snap frozen in liquid nitrogen, and stored at −80°C for zymography analysis. Tissue sections were cut from paraffin blocks and then stained with haematoxylin and eosin (H & E) as previously described [11].

### 2.2. Immunohistochemistry

Assessment of apoptotic proteins and MMP-9 was performed on formalin-fixed, paraffin-embedded tissue sections as we have previously described [11, 30, 31]. Briefly, once deparaffined and endogenous peroxide activity removed, sections of tissue were incubated for 1 h in antibody diluted in 1% BSA in TBS. Rabbit polyclonal anti-Bax (sc-526), rabbit polyclonal anti-Fas (sc-714), rabbit polyclonal anti-FasL (sc-957), mouse monoclonal anti-Bcl-2 (sc-509), and mouse monoclonal anti-Smac (sc-73039) were purchased from Santa Cruz (CA, USA) and used at 2 *μ*g/mL. Rabbit monoclonal anti-caspase-3 (9664) and rabbit polyclonal anti-PARP (9542) were purchased from Cell Signaling Technology (MA, USA) and used at 1 : 100. Rabbit polyclonal anti-MMP-9 (16996) was purchased from Millipore (Billerica, MA, USA) and used at 10 *μ*g/mL. After antibody incubation, the binding sites were labelled with Dako Envision + polymer linked secondary reagent and visualized using diaminobenzidine (DakoCytomation). Nuclei were counterstained with Mayer's haematoxylin, and the sections were dehydrated and coverslipped using a resinous mounting agent. Primary antibody was omitted in negative controls and replaced with normal rabbit or mouse IgG serum. Positive controls of tonsil, breast tumour, and ovarian tumour were included in each run. 

The entire tissue section was assessed microscopically for both intensity and extent of staining as we have previously described [11, 30, 31]. The evaluation of all immunohistochemical staining was done as a blind assessment and independently scored by an experienced pathologist (Clyde Riley) and by Mahalia Chai. The entire tissue section was scored, and the extent of staining was determined on a scale of 0–5 according to the estimated percentage of cells stained: 0 ≤ 10%; 1 = 11–25%; 2 = 26–50%; 3 = 51–75%; 4 = 76–90%, and 5 ≥ 90%. Staining intensity was assessed on a scale of 0–3: 0 = no staining, negative; 1 = pale brown, weak; 2 = brown, moderate; and 3 = dark brown, strong [32]. The intensity and extent of staining were averaged and used in the final analysis. 

### 2.3. Gelatin Zymography

Tissue samples (50 mg) were prepared as previously detailed [33]. Twenty micrograms of protein was added to an equal volume of zymography sample buffer (500 mM Tris-HCl, pH 6.8, 5% SDS, and 20% glycerol and bromophenol blue) loaded and separated on a 10% SDS-PAGE gel containing 0.1% (w/v) gelatin. Gels were incubated in prewarmed renaturation buffer (2.5% Triton X-100) three times for 20 min and then incubated for 48 h at 37°C in development buffer (200 mM NaCl, 50 mM Tris-HCl pH 7.6, 5 mM CaCl_2_, 1 *μ*M ZnCl_2_, and 0.02% (v/v) Triton X-100). Gels were stained with Coomassie Brilliant Blue dye (0.5% (w/v) Coomassie blue R250 in 30% methanol: 10% glacial acetic acid in water) and destained (50% methanol and 10% acetic acid in water). The enzyme activities were completely inhibited by heavy metal chelators added to the development buffer (1 mM of 1, 10-phenothroline, and 10 mM EDTA) to verify protease production (data not shown). Semiquantitative densitometry were performed on the inverted gel using Quantity One 4.2.1 image analysis software (Bio-Rad, USA).

### 2.4. Statistical Analysis

Statistical analyses were performed using Statgraphics Plus version 3.1 (Statistical Graphics Corp., USA). Data were normally distributed; therefore, no-labour SCS versus no-labour DS sample comparisons were analysed by paired sample comparison while no-labour SCS versus post-labour SOR were analysed by Student's *t*-test. Statistical significance was indicated by *P* value < 0.05. Data was expressed as mean ± SEM.

## 3. Results

### 3.1. General Morphologic Analysis

**Figure 1:**
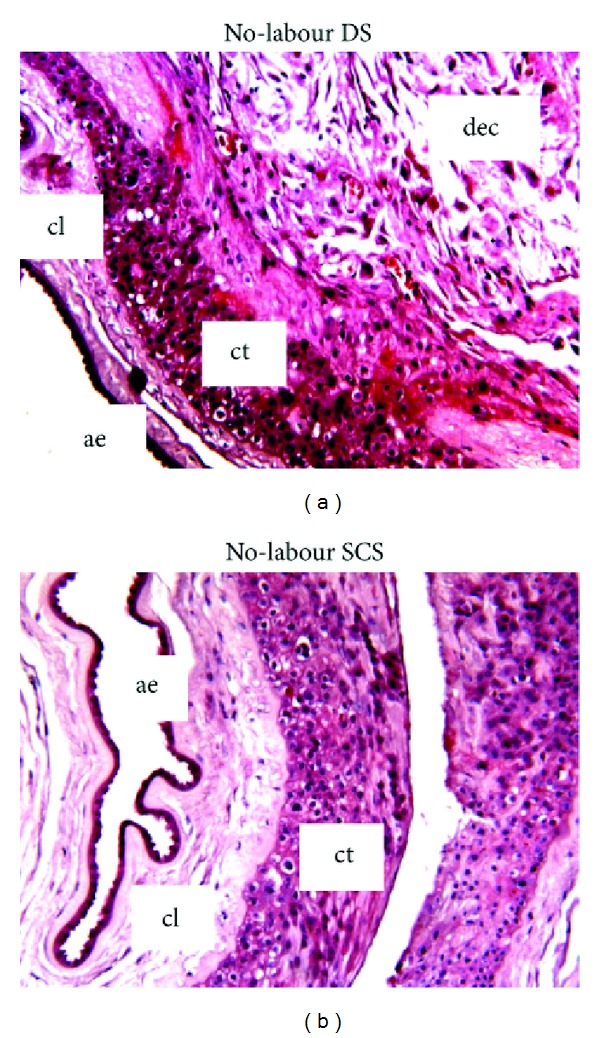
Hematoxylin and eosin stained sections of fetal membranes from the non-labouring DS, SCS. ae: amniotic epithelium; cl: connective tissue layer; ct: cytotrophoblast layer; and dec: decidua. Magnification 100x.

H & E was used to examine the general morphologic features of the fetal membranes obtained from the SCS and DS (Figure 1). When compared to the DS, the SCS fetal membranes consistently exhibited greater morphological alterations and greater thickness of the connective tissue layer and little decidua.

### 3.2. Expression of Apoptosis Pathway in Fetal Membranes

Immunohistochemistry was performed to determine the cellular localisation and expression of apoptotic proteins. Comparisons were made between no-labour SCS and paired DS fetal membranes and no-labour SCS versus post-labour SOR fetal membranes. The intensity and extent of staining of the proapoptotic intrinsic proteins, Bax and Smac, the extrinsic pathway antigens, Fas and FasL, the terminal apoptotic antigens are caspase-3 and PARP, and the anti-apoptotic protein Bcl-2 are presented in Table 2.

#### 3.2.1. Bax (Figure 2(a))

There was positive Bax staining which was mostly granular and perinuclear, in the SCS and DS amnion epithelium. SOR Bax staining in the amnion membranes was mostly granular. Granular, scattered Bax staining was also observed in trophoblast cells in chorion. The extent of Bax staining in the chorionic trophoblasts was significantly greater at the SCS than the DS; however, there was no difference in Bax expression between SCS and DS amnion epithelium. The extent of Bax staining was significantly greater in SCS compared to SOR fetal membranes.

#### 3.2.2. Smac (Figure 2(b))

There was positive Smac staining in the amnion epithelium and cytotrophoblast layer in the SCS which was predominantly cytoplasmic. The SOR exhibited positive, granular and cytoplasmic staining in both the amnion epithelium and chorionic cytotrophoblast layer. The intensity of Smac staining in the fetal membranes was significantly greater in the SCS compared to the DS. Additionally, in the chorionic trophoblast layer, the extent of staining was higher in the SCS compared to the DS. No difference in intensity and extent of Smac staining was observed between SCS and SOR fetal membranes. Decidua, where present, in the DS exhibited weak Smac staining.

#### 3.2.3. Fas (Figure 2(c))

Positive Fas staining was exhibited in the SCS and DS within the spongy layer of the fetal membranes and was mostly cytoplasmic. The extent of Fas staining was significantly greater in the SCS compared to the DS chorionic cytotrophoblast layer and no significant changes were noted in the amnion epithelium. The intensity and extent of Fas staining were not significantly different to the SCS when compared to the SOR.

#### 3.2.4. FasL (Figure 2(d))

Positive, mostly nuclear FasL staining was observed in the amnion epithelium and the chorionic cytotrophoblast layer. Positive FasL staining was also observed in the spongy layer. There was no difference in the intensity of staining at any site for any tissue. The extent of FasL staining was significantly greater in the SCS compared to the DS cytotrophoblast layer; however, no significant difference in the extent of FasL staining was seen between SCS and DS amnion epithelium. When compared to SOR chorion, the extent of FasL staining is significantly greater in the SCS chorionic cytotrophoblast layer, while no significant differences were observed in the amnion epithelium.

#### 3.2.5. Caspase-3 (Figure 2(e))

The SCS cytotrophoblast layer exhibited significantly greater extent of caspase-3 staining when compared to DS, which exhibited mostly cytoplasmic caspase-3 staining. The SOR exhibited positive caspase-3 staining, which was predominantly cytoplasmic. Fetal membranes exhibited caspase-3 staining in the SCS, which was significantly of greater intensity than the DS. In the cytotrophoblast layer, when compared to the SOR, the intensity of caspase-3 staining was significantly greater in the SCS. 

#### 3.2.6. PARP (Figure 2(f))

There was positive PARP staining which was mostly cytoplasmic in the SCS and DS. In the chorionic cytotrophoblast layer, the SCS exhibited significantly greater extent of PARP staining when compared to the DS. Furthermore, in the cytotrophoblast layer, PARP staining in the SCS was significantly more extensive when compared to the SOR, in which very little PARP staining was observed. 

#### 3.2.7. Bcl-2 (Figure 2(g))

Staining of the anti-apoptotic protein Bcl-2 was only observed in the decidua at the DS. No Bcl-2 staining was observed in the amnion epithelium and the chorionic cytotrophoblast layer.

**Figure 2:**
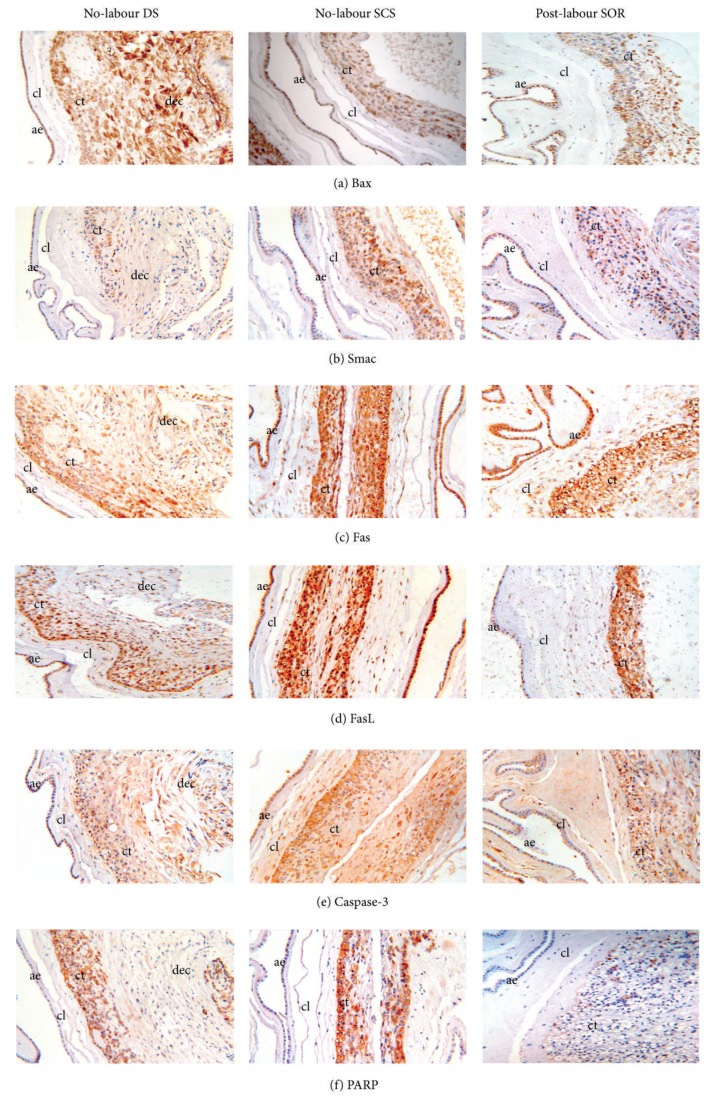

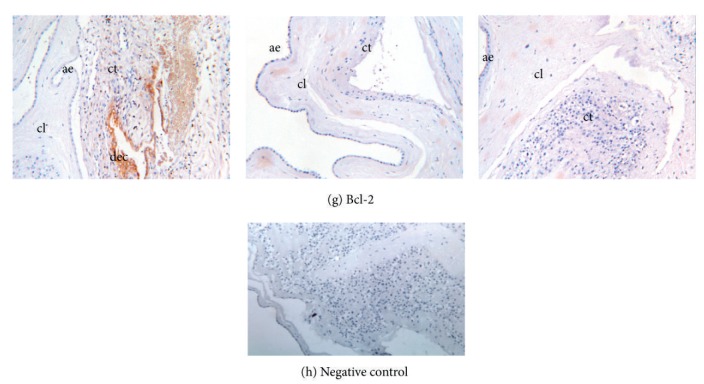
Immunohistochemical localisation of (a) Bax, (b) Smac, (c) Fas, (d) FasL, (e) caspase-3, (f) PARP, and (g) Bcl-2 in no-labour DS (left panel), no-labour SCS (middle panel), and post-labour SOR (right panel) fetal membranes. (h) Negative control. ae: amniotic epithelium; cl: connective tissue layer; ct: cytotrophoblast layer; and dec: decidua. Magnification 100x.

No staining was observed in the negative controls. A representative image is shown in Figure 2(h). Of note, staining for all proteins was observed in the positive controls of tonsil, breast tumour, and ovarian tumour.

### 3.3. Gelatinase Expression and Production in Fetal Membranes

**Figure 3:**
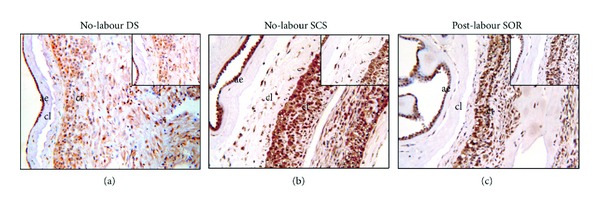
Immunohistochemical localisation of MMP-9 in (a) no-labour DS, (b) no-labour SCS, and (c) post-labour SOR fetal membranes. ae: amniotic epithelium; cl: connective tissue layer; ct: cytotrophoblast layer; and dec: decidua. Magnification 100x; magnification in insert 250x.

Table 3 is a summary of the intensity and extent of MMP-9 determined by immunohistochemistry in the fetal membranes. In the SCS MMP-9 staining was localised to the cytoplasm, and some positive MMP-9 staining was observed in the spongy layer (Figure 3). SOR had positive MMP-9 staining localised to the spongy, extracellular matrix with some cytoplasmic staining. No significant differences were observed in MMP-9 staining in SCS compared to the DS in both the amnion epithelium and the chorionic cytotrophoblast layer. SOR amnion epithelium exhibited moderately intense and extensive MMP-9 staining that was significantly greater compared with the SCS. 

**Figure 4:**
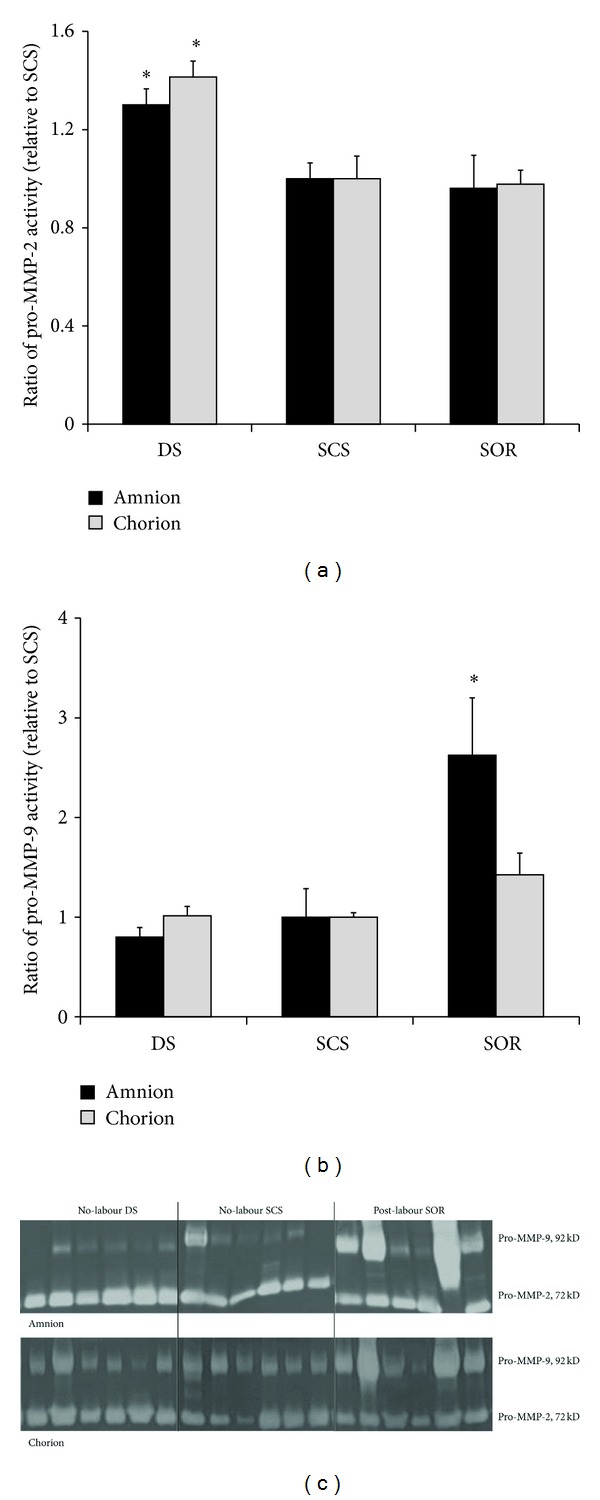
Effect of SCS apposition and labour, on (a) pro-MMP-2 and (b) pro-MMP-9 activities in fetal membranes. The bottom panel is the gelatin zymography demonstrating pro-MMP-2 and pro-MMP-9 expressions in amnion and chorion. Each bar represents the mean (relative to no-labour SCS) ± SEM (*n* = 6 patients per group). *P < 0.05 versus SCS (no-labour SCS versus no-labour DS analysed by paired sample comparison; no-labour SCS versus post-labour SOR analysed by Student's *t*-test).

Gelatin substrate zymography was used to characterise MMP activity in the fetal membranes.  Figure 4 shows the image of the zymogram of the pro-MMP-2 and pro-MMP-9 expressions in non-labour SCS and DS and post-labour SOR. Zymography revealed lysis bands at approximately 72 kD, corresponding to pro-MMP-2 and another band at 92 kD corresponding to pro-MMP-9. In some samples, the active MMP-9 band was also present. Inhibition by heavy metal chelators confirmed that these bands were MMPs (data not shown). When compared to SCS, pro-MMP-2 production in both the amnion and chorion was significantly greater in the DS. SOR pro-MMP-9 production was greater when compared to the SCS, although it was only significant in the amnion.

## 4. Discussion

In this study, fetal membranes display both regional and labour morphological changes including diminution of the decidua and increased connective tissue at the SCS and the SOR. When compared to DS, the extent of pro-apoptotic markers at the no-labour SCS was increased, particularly at the SCS chorion. Furthermore, when compared to post-labour SOR, the no-labour SCS exhibited greater protein expression in some of the pro-apoptotic markers. MMP-9 protein expression was greater at the SOR compared to SCS. Moreover, pro-MMP-2 expression was greater in fetal membranes from the DS than that from the matched SCS.

Fetal membrane sections from the SCS and SOR exhibited marked disruption and swelling of connective tissue. Maternal decidua is observed at the DS, a marked contrast to the SCS which lacked decidua or if present the decidua appeared to be markedly thinner compared to the DS. These morphological observations are consistent with other studies [12, 15, 34]. This suggests that the SCS represents an area of compromised structural integrity, susceptible to rupture during labour. Additionally, as previously reported [7], we observed that the general morphology of non-labouring SCS fetal membranes was similar to post-labour SOR, suggesting that these morphological changes predate labour.

The altered morphologic characteristics that occur in the SCS fetal membranes have been associated with MMP production and apoptosis [9, 11, 15, 20, 34]. Previously, the intrinsic apoptotic pathway has been shown to be the dominant pathway in the combined amniochorion SCS when compared to DS [11]. In the present study, we found that both intrinsic and extrinsic pathways were present in the fetal membranes. Of note, we found that the expression of the six proapoptotic markers we studied (Bax, Smac, Fas, FasL, caspase-3, and PARP) was higher in the SCS compared to the DS chorion. This is consistent with previous studies, which have found that the SCS has an increase in apoptotic bodies [14] and PARP cleavage [9] compared to regions that are more distal. Increased apoptosis at the SCS at term supports the hypothesis that there is programmed weakening of the fetal membranes at the SCS in preparation for rupture. This is consistent with our observation which has been reported by us [11] and others [20] that there was no anti-apoptotic protein, Bcl-2, in SCS or SOR fetal membranes and yet was found in the DS at the decidua. PPROM and PROM membranes have been demonstrated to show an increase of pro-apoptotic p53 and Bax gene expressions and a decrease in the anti-apoptotic Bcl-2 gene [35]. The anti-apoptotic protein Bcl-2 expression in the decidua and its role in fetal membrane rupture are unknown, but it is tempting to speculate that the anti-apoptotic regulation by Bcl-2 in the decidua may be the reason that unregulated apoptotic activity is seen at the SCS chorion, resulting in intrinsic weakness and thereby facilitating weakening and eventual rupture at the SCS.

It is speculated that the separation of the amnion and chorion is thought to be an important step towards membrane rupture [36]; subsequently this report examined the membranes layers independently. Biophysical studies have shown that the chorion ruptures first. This is consistent with our findings of greater expression of pro-apoptotic markers in the SCS chorion. Moreover, the extent of apoptotic staining was greater in chorion from the no-labour SCS compared to post-labour chorion from the SOR. This supports the hypothesis that there is programmed weakening of the fetal membranes at the SCS in preparation for labour.

In the rat fetal membranes as term approaches, MMPs increase which corresponds to a subsequent decrease in collagen [4–6]. MMP-9 expression and pro-MMP-9 production were significantly increased in the labouring SOR amnion compared to the no-labour SCS. We suggest that MMP-9 may be a major contributor in membrane rupture during labour at term where increased MMP-9 may result in degradation of the ECM. This is consistent with other data on MMP-9, where it has been observed to be increased in spontaneous parturition, preterm labour, PROM, PPROM, and infection in amniotic fluid [22, 25–27, 37–41]. Fetal plasma MMP concentrations are higher in fetuses with PPROM compared to those with preterm labour [42], and maternal gene DNA variants in the genes involved in ECM metabolism double the risks of PPROM [43]. Tissue inhibitors of metalloproteinases (TIMP) alter with labour (preterm and term), PROM, and PPROM in the amniotic fluid [40, 41, 44–46]. Previous studies have found that the SCS exhibits decreased TIMP-3 protein [9] and the SOR exhibits decreased TIMP-3 [8]. In this study, we were unable to detect MMP-2 protein expression; despite this, zymography revealed that pro-MMP-2 production was significantly greater in DS amnion and chorion compared to the SCS. There was no significant difference in MMP-2 expression or pro-MMP-2 expression between SCS and SOR. MMP-2 has been detected in fetal membranes early in gestation, is constitutive, and does not change with labour, PROM, or PPROM and does not respond to cytokines [26, 40, 41, 47]. The exact regional role of MMP-2 in the fetal membranes is unknown; however, MMP-2 may play an important role in remodeling during pregnancy with the growing uterine contents [25, 48]. Collectively, the data supports a role for MMP-9 in the preparation of labour and its role in controlling the degradation of the ECM.

There are many inducers of MMPs and apoptosis. Lipopolysaccharide (LPS), the pro-inflammatory cytokines IL-1*β* and TNF-*α*, and thrombin all increase MMP-9 and/or PARP expression in human fetal membranes [49–53]. Cigarette smoke has been found to induce a dose-dependent decrease in Bcl-2 expression and increase caspase-3 activity [54]. All these studies implicate apoptosis and MMPs in the pathophysiology of rupture of fetal membranes, at both term and preterm. We are currently undertaking continuing studies in our laboratory expanding our understanding of inducers and inhibitors by examining SCS and DS response of various inducers of apoptosis and MMPs. Understanding the factors that may attenuate fetal membrane rupture will have substantial benefits associated with preventing and treating PROM and PPROM.

Part of the reason for the inability to prevent PROM and PPROM may be attributed to the lack of understanding of basic molecular mechanisms underlying fetal membrane rupture. This paper provides insights into the heterogeneous and temporal apoptotic and MMP pathways in the fetal membranes. The data presented in this study confirm that the SCS in fetal membranes before labour is morphologically and biochemically different to the DS. Apoptosis may have a causal role in the degeneration changes at the SCS in periparturitional membranes. Additionally, MMP-9 expression and production were found to significantly increase after labour, indicating labour-associated changes. A coherent picture of how apoptosis and MMPs are regulated and executed with regard to regional differences and the impact of labour may lead to the development of preventive strategies to minimise weakening or improved strategies for membrane repair. 

## Figures and Tables

**Table 1:** Patient characteristics.

Clinical characteristic	No-labour (*n* = 6)	Post-labour (*n* = 6)
Maternal age (years)	32.8 ± 1.1	31.0 ± 0.4
Maternal BMI (kg/m^2^)^#^	25.0 ± 1.1	23.2 ± 1.1
Gravida	4.1 ± 0.7	2.8 ± 0.4
Parity	3.1 ± 0.5	2.0 ± 0.3*
Gestation age (weeks)	38.5 ± 0.6	37.8 ± 0.7
Birthweight (grams)	3426.4 ± 125.3	3405.8 ± 125.8
Gender of infant (%)	58 male; 42 female	50 male; 50 female

*P < 0.05 versus no-labour (Student's *t*-test).

^#^Approx. 12 weeks of gestation.

**Table 2:** Intensity and extent of staining of apoptotic markers from non-labouring and post-labour SC and distal fetal membranes.

	Antigen	Tissue	Intensity of staining			Extent of staining		
			No-labour		Post-labour	No-labour		Post-labour
			DS	SCS	SOR	DS	SCS	SOR
Intrinsic pathway	Bax	Amnion	1.8 ± 0.2	1.7 ± 0.2	1.2 ± 0.2	3.2 ± 0.0	3.8 ± 0.2	1.8 ± 0.2*
		Chorion	2.0 ± 0.0	2.0 ± 0.0	2.0 ± 0.4	3.2 ± 0.4*	4.2 ± 0.3	2.5 ± 0.2*
	Smac	Amnion	0.0 ± 0.0*	0.8 ± 0.2	0.8 ± 0.8	0.0 ± 0.0	0.7 ± 0.3	0.8 ± 0.9
		Chorion	1.0 ± 0.0*	1.7 ± 0.2	1.5 ± 0.2	1.8 ± 0.4*	3.5 ± 0.2	2.3 ± 0.6

Extrinsic pathway	Fas	Amnion	1.8 ± 0.2	1.8 ± 0.2	1.8 ± 0.2	4.3 ± 0.2	3.8 ± 0.3	4.0 ± 0.3
		Chorion	3.3 ± 0.3	2.0 ± 0.5	2.0 ± 0.2	3.5 ± 0.3*	4.3 ± 0.2	4.0 ± 0.4
	FasL	Amnion	2.0 ± 0.0	2.0 ± 0.3	2.0 ± 0.0	1.7 ± 0.2	1.8 ± 0.5	2.5 ± 0.4
		Chorion	2.0 ± 0.0	2.5 ± 0.2	2.0 ± 0.2	3.0 ± 0.0*	4.0 ± 0.3	3.0 ± 0.3*

Common terminal pathway	Caspase-3	Amnion	0.0 ± 0.0*	0.7 ± 0.2	0.5 ± 0.2	0.0 ± 0.0	0.5 ± 0.2	0.7 ± 0.3
		Chorion	1.0 ± 0.0*	1.7 ± 0.2	1.0 ± 0.0*	1.8 ± 0.3*	3.5 ± 0.2	2.7 ± 0.5
	PARP	Amnion	0.0 ± 0.0	0.0 ± 0.0	0.0 ± 0.0	0.0 ± 0.0	0.0 ± 0.0	0.0 ± 0.0
		Chorion	2.0 ± 0.0	1.8 ± 0.3	1.2 ± 0.2	3.0 ± 0.3*	4.0 ± 0.3	0.8 ± 0.3*

Antiapoptotic	Bcl-2	Amnion	0.0 ± 0.0	0.0 ± 0.0	0.0 ± 0.0	0.0 ± 0.0	0.0 ± 0.0	0.0 ± 0.0
		Chorion	0.0 ± 0.0	0.17 ± 0.2	0.0 ± 0.0	0.0 ± 0.0	0.0 ± 0.0	0.0 ± 0.0

All data is expressed as mean ± SEM (*n* = 6 patients per group).

*P < 0.05 versus SCS (no-labour SCS versus no-labour DS analysed by paired sample comparison; no-labour SCS versus post-labour SOR analysed by Student's *t*-test).

**Table 3:** Intensity and extent of staining of MMP-9 in non-labouring and post-labour SC and distal fetal membranes.

	Intensity of staining			Extent of staining		
Tissue	No-labour		Post-labour	No-labour		Post-labour
	DS	SCS	SOR	DS	SCS	SOR
Amnion	1.7 ± 0.2	2.0 ± 0.3	3.2 ± 0.2*	2.8 ± 0.5	2.8 ± 0.5	4.3 ± 0.2*
Chorion	2.0 ± 0.0	2.2 ± 0.2	2.7 ± 0.2	4.0 ± 0.3	4.3 ± 0.2	4.7 ± 0.2

All data is expressed as mean ± SEM (*n* = 6 patients per group).

*P < 0.05 versus SCS (Student's *t*-test).
